# Deep Learning-Based Retinal Vascular Analysis with Quantitative Metrics for Hypertensive Microvascular Damage

**DOI:** 10.3390/jcm15218715

**Published:** 2026-11-08

**Authors:** Ceren Durmaz Engin, Efekan Bilen, Barbaros Hayrettin Unlu, Mustafa Alper Selver, Andrzej Grzybowski

**Affiliations:** 1 Department of Ophthalmology, İzmir Democracy University, 35330 Izmir, Turkey; ceren.engin@idu.edu.tr; 2 Artificial Intelligence and Data Analytics Application & Research Center, Izmir Democracy University, 35140 Izmir, Turkey; 3 Department of Electrics and Electronics, Dokuz Eylül University, 35390 Izmir, Turkey; efekanbilenn@gmail.com (E.B.); alper.selver@deu.edu.tr (M.A.S.); 4 Department of Ophthalmology, Menemen State Hospital, 35660 Izmir, Turkey; barbarosunlu621@gmail.com; 5 Department of Ophthalmology, University of Warmia and Mazury, 10-719 Olsztyn, Poland; 6 Institute for Research in Ophthalmology, Foundation for Ophthalmology Development, 61-553 Poznan, Poland

**Keywords:** hypertension, hypertensive retinopathy, hypertension-mediated target organ damage, retinal vascular analysis, deep learning, retinal microvasculature

## Abstract

**Background/Objectives**: The goal of this study was to determine whether deep learning (DL)-assisted retinal vascular analysis detects subclinical microvascular remodeling associated with hypertension-mediated target organ damage (TOD) in patients with absent or mild hypertensive retinopathy (HR). **Methods**: This prospective study included age-comparable hypertensive patients with TOD (*n* = 54), hypertensive patients without TOD (*n* = 54), and healthy controls (*n* = 54). Color fundus photographs underwent DL-based segmentation using the Retinal Microvascular Health Assessment System, ophthalmologist quality review, and Fiji morphometric analysis. Central retinal arteriolar and venular equivalents (CRAE and CRVE), arteriovenous ratio (AVR), and arterial and venous network metrics were quantified. **Results**: AVR decreased progressively from 0.814 ± 0.038 in controls to 0.740 ± 0.043 in hypertensive patients without TOD and 0.655 ± 0.042 in those with TOD (*p* < 0.001). CRAE and CRVE differed among groups (*p* < 0.001 and *p* = 0.002). Arterial fractal dimension and vessel area density (VAD) declined stepwise (both *p* < 0.001), whereas arterial skeletonized vascular density (SVD) and diameter were primarily reduced in TOD. Venous VAD and diameter differed (*p* = 0.022 and *p* = 0.006), but venous SVD did not (*p* = 0.146). After adjustment among hypertensive participants, TOD remained associated with lower AVR, CRAE, and all arterial network metrics except tortuosity (all *p* < 0.001), but not with venous parameters. **Conclusions**: Retinal arterial alterations were associated with established extra-retinal TOD in patients with absent or mild HR. Residual cardiovascular confounding limits interpretation, and longitudinal studies are needed to establish temporal relationships, predictive value, and clinical utility.

## 1. Introduction

Hypertensive retinopathy (HR) is a chronic microvascular manifestation of systemic hypertension (HT) and serves as a unique in vivo marker of hypertensive microvascular injury. Because the retinal vasculature shares anatomical and physiological characteristics with the cerebral and coronary microcirculation, retinal examination provides a non-invasive surrogate marker of systemic vascular health. Early and accurate assessment of hypertensive retinal microvascular changes is clinically important because these alterations are associated with an increased risk of stroke, cardiovascular disease, and mortality. However, current HR assessment still relies largely on qualitative grading systems, such as the Keith–Wagener–Barker classification [1,2]. These systems primarily identify established disease and have limited sensitivity for detecting early and subtle vascular alterations, including arteriolar narrowing, venular dilation, and reduced vascular complexity, while also being susceptible to inter-observer variability.

Quantitative retinal vascular metrics have emerged as objective imaging biomarkers to overcome the limitations of qualitative HR grading. Established caliber-based parameters, including central retinal arteriolar equivalent (CRAE), central retinal venular equivalent (CRVE), and arteriovenous ratio (AVR), have been validated in large population-based studies and shown to be associated with HT and future cardiovascular risk [1,3]. Network-level descriptors such as fractal dimension, vessel area density (VAD), skeletonized vascular density (SVD), and tortuosity have been used to characterize vascular complexity and integrity, with reduced fractal dimension and VAD reflecting microvascular rarefaction and early vascular damage [4]. However, clinical translation has been limited by the need for accurate vessel segmentation and standardized post-processing, as fully manual or semi-automated workflows remain time-consuming, operator-dependent, and vulnerable to measurement variability.

Advances in deep learning (DL), particularly U-Net-based architectures, have enabled automated vessel segmentation, artery–vein classification, optic disc detection, and quantitative metric extraction. Integrated systems, such as the multi-branch U-Net framework proposed by Shi et al. [5], now allow reproducible measurement of CRAE, CRVE, and AVR, while end-to-end models have demonstrated the ability to predict cardiovascular risk factors directly from retinal images [4,6,7]. Despite these advances, an important limitation of previous retinal vascular studies is the way clinical phenotypes have been defined. Many studies have relied on broad binary classifications, such as the presence or absence of HT or cardiovascular disease, which may not adequately reflect the continuous and progressive nature of hypertensive microvascular injury [8,9,10]. In addition, although automated segmentation techniques have significantly improved retinal image analysis, segmentation errors may still occur and can lead to inaccurate vascular measurements if not carefully reviewed and excluded.

In this context, the present study aimed to determine whether DL-assisted quantitative retinal vascular metrics can identify subclinical hypertensive microvascular alterations in patients with absent or mild HR. Unlike previous studies, which primarily relied on binary clinical classifications or fully automated image analysis, we stratified hypertensive patients according to the presence or absence of target organ damage (TOD) and manually validated automated artery–vein segmentation before quantitative vascular analysis.

## 2. Materials and Methods

### 2.1. Study Design and Study Population

This prospective observational study, conducted at a tertiary university hospital from 1 March to 1 July 2026, included three groups with comparable mean ages: hypertensive patients with or without TOD and healthy controls. The TOD group consisted of patients with systemic HT for at least five years and evidence of TOD other than the mild HR permitted for study inclusion, defined according to the 2023 European Society of Hypertension Guidelines endorsed by the International Society of Hypertension and the European Renal Association [11]. TOD was considered present when at least one of the following was documented: urinary albumin-to-creatinine ratio (UACR) ≥ 30 mg/g, estimated glomerular filtration rate of 30–59 mL/min/1.73 m^2^, left ventricular hypertrophy on electrocardiography or echocardiography, carotid intima–media thickness > 0.9 mm, or carotid plaque. The second group included patients with systemic HT for at least five years but without evidence of non-retinal (renal, cardiac, or major vascular) TOD. Healthy controls had no history of HT, office blood pressure < 130/80 mmHg, no evidence of systemic TOD, and no retinal vascular pathology.

Eligible participants were adults aged 18–80 years who, if included in the hypertensive groups, had received stable antihypertensive treatment for at least four weeks before imaging. Routine clinical records documented controlled hypertension and recommended continuation of the existing antihypertensive regimen. However, numerical blood-pressure readings contemporaneous with retinal imaging were not available for analysis, precluding a standardized assessment of blood-pressure control at the imaging visit.

Exclusion criteria were diabetes mellitus, retinal vascular disease other than HR, retinal degenerative disease, significant media opacity, glaucoma, uveitis, high refractive error (spherical equivalent > +3.0 D or <−3.0 D), pathologic myopia, retinal surgery within the previous six months, acute systemic illness, and pregnancy.

Image-quality exclusions involved artifacts, suboptimal centration, or blurring consistent with possible media opacity. Segmentation-related exclusions mainly involved incomplete vessel delineation within the peripapillary measurement annulus, associated with peripapillary changes or low vessel-to-background contrast, preventing reliable CRAE and CRVE estimation.

**Figure 1:**
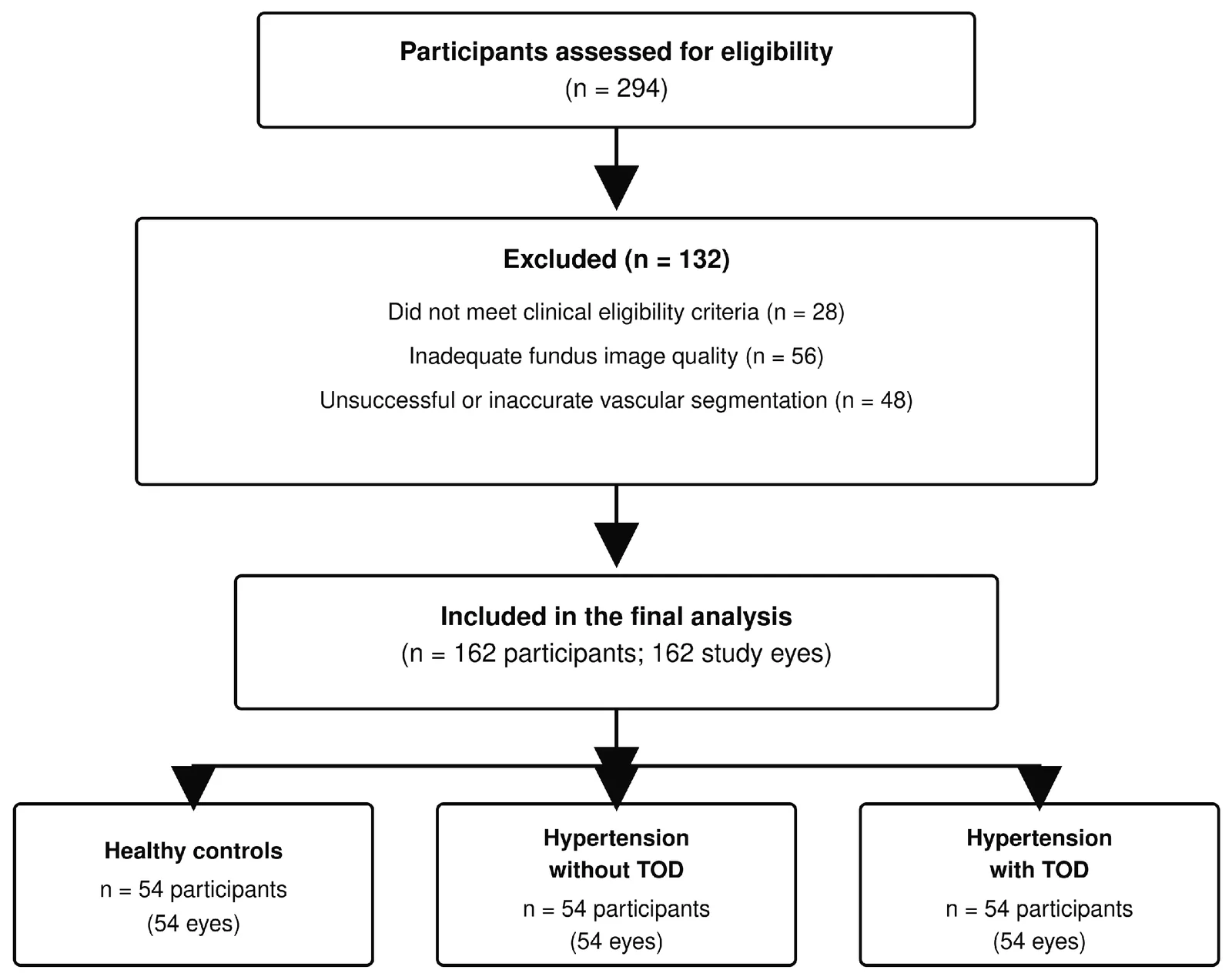
Flow diagram of participant selection. Of the 294 participants assessed for eligibility, 28 did not meet the clinical eligibility criteria, 56 were excluded because of inadequate fundus image quality, and 48 were excluded because of unsuccessful or inaccurate vascular segmentation. The final analysis included 162 participants contributing one study eye each, with 54 participants in each group.

One eye per participant was analyzed to avoid within-subject correlation. When both eyes were eligible, the study eye was selected using computer-generated randomization; otherwise, the eligible eye was included. Eligibility required a gradable color fundus photograph, successful automated vascular segmentation, and no or mild HR. Figure 1 summarizes participant selection and exclusions for clinical ineligibility, poor image quality, or segmentation failure.

The study protocol was approved by the Institutional Review Board of Buca Seyfi Demirsoy Education and Research Hospital (Approval No. 2026/124; 25 February 2026). The study adhered to the principles of the Declaration of Helsinki, and written informed consent was obtained from all participants before enrollment.

### 2.2. HR Status

Hypertensive retinopathy was graded according to the simplified Wong and Mitchell classification, which categorizes retinal changes as none, mild, moderate, or malignant [12]. Because mild HR lacks well-defined objective diagnostic criteria, grading was performed independently by two ophthalmologists. Mild HR was defined by the presence of generalized or focal arteriolar narrowing, arteriovenous nicking, increased arteriolar wall reflectivity, or any combination of these findings.

Healthy controls had no clinical signs of HR. Eyes with retinal hemorrhages, hard exudates, cotton-wool spots, optic disc edema, or other features consistent with moderate or malignant HR were excluded. Eligibility required agreement between two ophthalmologists that the fundus findings were consistent with no or mild HR, without moderate or malignant changes. Participants for whom consensus regarding mild HR could not be reached were excluded on clinical eligibility grounds.

### 2.3. Fundus Image Acquisition and Deep Learning-Based Retinal Vessel Segmentation

Macula-centered color fundus photographs were acquired using a swept-source optical coherence tomography-based fundus imaging system (Topcon DRI OCT Triton; Topcon Corporation, Tokyo, Japan) under standardized imaging conditions. All photographs were obtained using the same imaging system and acquisition protocol and were exported as 24-bit color images at a native resolution of 2592 × 1728 pixels. Images were reviewed before analysis, and only photographs with adequate focus, illumination, contrast, and retinal field definition were included. Images with motion artifacts, insufficient image quality, media opacity obscuring the retinal vessels, or failed vascular segmentation were excluded.

Images were processed using the publicly available Retinal Microvascular Health Assessment System (RMHAS) described by Shi et al. [5] (available from https://www.retinavessel.com; access date: during the study period of 1 March–1 July 2026). RMHAS uses a multibranch U-Net to segment the optic disc, arteries, and veins. All RMHAS outputs were reviewed by two ophthalmologists for optic disc localization, vessel segmentation quality, and artery–vein classification accuracy. Segmentation failure was defined as inaccurate optic disc localization, incomplete visualization of the required measurement region, major vessel discontinuity or omission, false-positive segmentation of nonvascular structures that could materially affect quantitative measurements, or artery–vein misclassification involving vessels used for caliber analysis. Images meeting any of these criteria were excluded, and no manual correction of the segmentation masks was performed. Disagreements between the ophthalmologists were resolved by consensus.

Although the photographs were macula-centered, caliber measurements were accepted only when the optic disc and the complete standard measurement annulus were visible within the retinal field. According to the RMHAS protocol, this standard zone was defined as the annular region extending from 0.5 to 1.0 optic disc diameter from the optic disc margin. The six largest arteries and six largest veins crossing this zone were used for the automated calculation of CRAE and CRVE. The AVR was calculated as CRAE divided by CRVE.

### 2.4. Fiji-Based Quantitative Retinal Vascular Analysis

The RMHAS-generated color-coded segmentation images, in which arteries and veins were labeled in red and blue, respectively, were imported into Fiji (ImageJ version 1.54t; National Institutes of Health, Bethesda, MD, USA; https://fiji.sc). The red channel was extracted to generate the arterial map, whereas the blue channel was extracted to generate the venous map. Each channel was converted to 8-bit grayscale, and intensity thresholding was applied within the 0–255 range to retain the labeled vessels and optic disc. The thresholded images were subsequently converted into binary masks.

Because the optic disc was also retained during thresholding, its boundary was manually delineated using Fiji’s Oval Selection tool and removed from each binary mask. The resulting masks therefore contained only vessel-positive pixels. The same procedure was applied separately to the arterial and venous maps.

Vessel area density was measured using the Analyze > Measure command and was defined as the number of white vessel pixels divided by the total number of pixels in the standardized image.

The binary vascular maps were subsequently reduced to one-pixel-wide centerlines using Fiji’s Skeletonize command. Skeletonized vascular density was defined as the number of skeleton pixels divided by the total number of image pixels. Average vessel diameter was measured separately for the arterial and venous networks using Fiji’s Local Thickness (Masked, Calibrated, Silent) plug-in, which assigns to every vessel pixel the diameter of the largest inscribed sphere fitting within the binary mask at that location. The thickness map was masked with the corresponding skeleton, and the mean of the skeleton-restricted thickness values was taken as the image-level average vessel diameter, expressed in pixels. Because diameter was derived from the local thickness map rather than from the ratio of the two density measures, it constitutes an independent morphometric parameter and is not algebraically determined by VAD and SVD.

Arterial tortuosity was calculated after skeletonization using Fiji’s Analyze Skeleton (2D/3D) function. Individual branch segments were defined as vascular paths between successive endpoints and/or bifurcation points. For each branch, tortuosity was calculated as the ratio of the actual centerline path length to the Euclidean distance between its endpoints. The arithmetic mean of all branch-level measurements was used as the image-level arterial tortuosity value.

Arterial fractal dimension was calculated using Analyze > Tools > Fractal Box Count. Box sizes of 2, 3, 4, 6, 8, 12, 16, 32, and 64 pixels were used. Identical settings were applied to all arterial masks.

AVR, fractal dimension, vascular tortuosity, VAD, and SVD are reported as dimensionless image-derived parameters. CRAE, CRVE, and average vessel diameter are reported in algorithm-space pixels (px). All images were acquired using the same imaging system and processed at the same standardized resolution. Because axial-length measurements were unavailable, individual ocular magnification correction was not performed, and pixel-based caliber measurements were not converted to micrometers. Accordingly, these measurements represent relative image-derived estimates suitable for standardized within-study group comparisons rather than absolute anatomical vessel dimensions.

All Fiji-based analyses were performed jointly by two investigators blinded to clinical group and TOD status. The optic-disc boundary was delineated by consensus, and subsequent processing used the standard Fiji functions and plug-ins described above. As measurements were generated jointly rather than independently, interobserver intraclass correlation coefficient could not be calculated. Intraobserver reproducibility was not formally assessed.

**Figure 2:**
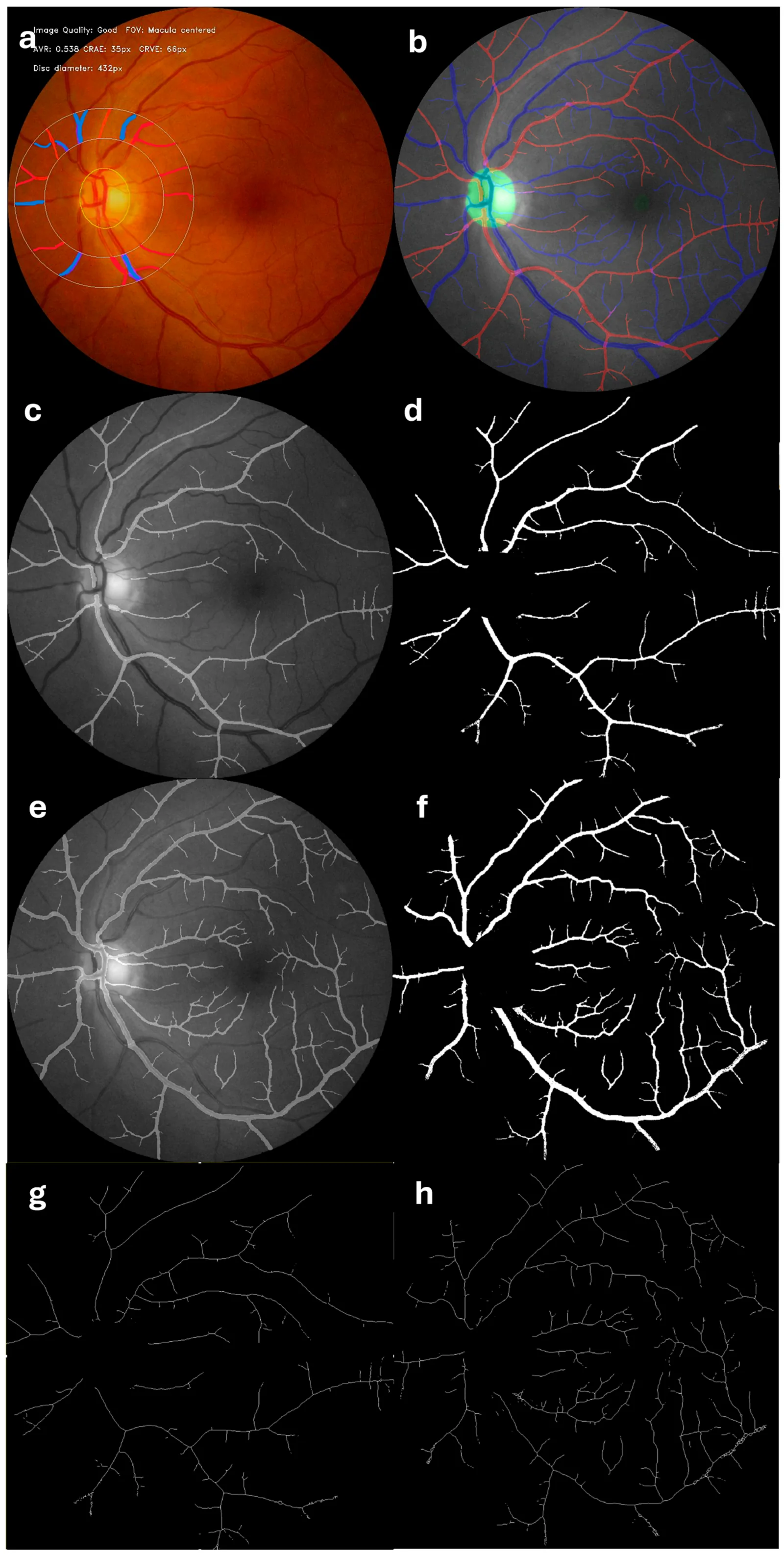
Workflow of retinal vascular image processing and quantitative vascular analysis. (**a**) Automated artery–vein classification within the optic disc-centered measurement zone for calculation of the arteriovenous ratio (AVR). (**b**) Automated artery–vein classification across the entire retinal image (arteries, red; veins, blue). (**c**,**e**) Grayscale arterial and venous vascular maps. (**d**,**f**) Corresponding binary vessel masks used for quantitative vascular analysis. (**g**,**h**) Skeletonized arterial and venous vascular maps used for the calculation of skeletonized vascular density, fractal dimension (art), and vascular tortuosity (art).

Figure 2 illustrates the image processing workflow, from automated artery–vein classification to grayscale conversion, vessel segmentation, and generation of artery-only and vein-only vascular maps for quantitative analysis.

### 2.5. Statistical Analysis

Assuming a moderate effect size (Cohen’s f = 0.25), a significance level of 0.05, and 80% statistical power, a minimum of 53 participants per group (159 participants in total) was required for a one-way ANOVA comparing three equally sized groups.

Statistical analyses were performed using Python 3.12.3 with SciPy 1.17.1, NumPy 2.4.4, and pandas 3.0.2. Continuous variables were presented as mean ± standard deviation and categorical variables as counts and percentages. Three-group comparisons were performed using one-way analysis of variance, and post hoc pairwise comparisons were performed using Welch’s *t*-test with Bonferroni correction for three comparisons. Comparisons across different vascular endpoints were considered exploratory, and no additional across-endpoint multiplicity correction was applied.

Duration of HT, which was recorded only in the two hypertensive groups, was compared using Welch’s *t*-test. Categorical variables were compared using the chi-square test; as no expected cell count was below five, Fisher’s exact test was not required.

Retinal vascular parameters were additionally evaluated using multivariable linear regression fitted by ordinary least squares. Full-cohort models included study group, age, and sex, with healthy controls as the reference category; models restricted to hypertensive participants also included duration of HT, with hypertensive patients without TOD as the reference category. Current blood pressure, degree of blood-pressure control, antihypertensive medication classes, smoking status, BMI, and dyslipidemia were not available for analysis and therefore could not be included as covariates.

Overall group effects were assessed using partial F-tests, and results were reported as unstandardized β coefficients with 95% confidence intervals derived from the t-distribution. Model assumptions were assessed through residual inspection. As only one eye per participant was included, adjustment for inter-eye correlation was unnecessary. All tests were two-sided, with *p* < 0.05 considered statistically significant.

## 3. Results

A total of 162 participants, contributing 162 study eyes, were included in the final analysis, with 54 eyes in each group. Of 294 participants assessed, 28 were clinically ineligible, 56 were excluded for inadequate image quality, and 48 for segmentation failure, leaving 162 participants for analysis. Image-quality exclusions numbered 22, 16, and 18, and segmentation-related exclusions numbered 13, 18, and 17 in healthy controls, HT without TOD, and HT with TOD, respectively. Overall image-related exclusion rates were similar across groups (39.3%, 38.6%, and 39.3%; *p* = 0.994). Mean ages of excluded participants were 59.2, 60.1, and 60.8 years, with male proportions of 45.7%, 58.8%, and 54.3%, respectively. Overall, excluded participant ts were descriptively older than included participants (approximately 60.0 versus 58.2 years), while sex distribution did not differ significantly (52.9% versus 48.1% male; *p* = 0.451).

The groups of included population were comparable in terms of age and sex distribution (*p* = 0.251 and *p* = 0.929, respectively). Patients with HT and TOD had a longer duration of HT, higher UACR, lower estimated glomerular filtration rate, and greater carotid intima–media thickness (all *p* < 0.001). Carotid plaque and left ventricular hypertrophy were observed exclusively in the HT with TOD group. Baseline demographic and systemic characteristics are presented in Table 1.

Among the 54 participants with TOD, 38 (70.4%) had UACR ≥ 30 mg/g, 42 (77.8%) had eGFR of 30–59 mL/min/1.73 m^2^, 24 (44.4%) had left ventricular hypertrophy, 37 (68.5%) had carotid intima–media thickness > 0.9 mm, and 22 (40.7%) had carotid plaque. One, two, and three organ systems were affected in 16 (29.6%), 20 (37.0%), and 18 (33.3%) participants, respectively. Coexisting abnormalities within the same organ system were counted as a single organ involvement.

Quantitative retinal vascular caliber measurements differed significantly among the groups (Table 2). AVR progressively decreased from 0.814 ± 0.038 in healthy controls to 0.740 ± 0.043 in patients with HT without TOD and 0.655 ± 0.042 in patients with HT and TOD (*p* < 0.001), with significant differences between all groups. CRAE was significantly lower in the HT with TOD group than in the other two groups (*p* < 0.001), whereas healthy controls and patients with HT without TOD did not differ significantly. CRVE also differed among the groups (*p* = 0.002) and was highest in patients with HT without TOD. Pairwise comparisons are presented in Table 3.

Arterial network metrics showed progressive alterations across the study groups. Arterial fractal dimension and VAD decreased significantly across all three groups (overall *p* < 0.001). Arterial SVD and vessel diameter were significantly lower in the HT with TOD group than in the other two groups. Arterial tortuosity showed a modest overall difference (*p* = 0.014), primarily reflecting greater tortuosity in the HT with TOD group than in healthy controls.

Venous VAD increased across the groups and was significantly higher in the HT with TOD group than in healthy controls (overall *p* = 0.022). Venous vessel diameter also differed among the groups (*p* = 0.006), with the highest value observed in patients with HT without TOD. In contrast, venous SVD did not differ significantly (*p* = 0.146).

After adjustment for age and sex, the overall group effect remained significant for all retinal parameters except venous SVD (Table 4). The HT without TOD group was characterized predominantly by lower AVR and arterial network metrics, together with higher CRVE and venous vessel diameter. The HT with TOD group showed more pronounced reductions in AVR, CRAE, and arterial network metrics, as well as greater arterial tortuosity and venous VAD, compared with healthy controls.

In analyses restricted to hypertensive participants and additionally adjusted for HT duration, TOD remained independently associated with lower AVR, CRAE, arterial fractal dimension, arterial VAD, arterial SVD, and arterial vessel diameter (all *p* < 0.001; Table 5). CRVE, arterial tortuosity, and venous network parameters were not independently associated with TOD status. Hypertension duration was not independently associated with any retinal vascular parameter.

## 4. Discussion

The present study demonstrated that DL-assisted quantitative retinal vascular analysis identified predominantly arterial retinal microvascular alterations associated with extra-retinal target organ damage in patients with absent or mild HR. AVR, arterial fractal dimension, and arterial VAD showed stepwise reductions across healthy controls, patients with HT without TOD, and patients with HT and TOD. Several additional arterial parameters were primarily altered in the HT with TOD group, and these associations persisted after adjustment for age, sex, and HT duration. These findings extend previous population-based studies showing that retinal vascular measurements reflect systemic microvascular injury and suggest that quantitative retinal biomarkers may detect hypertensive vascular remodeling despite absent or mild HR [1,3,13]. By contrast, venous findings were less consistent and did not independently distinguish hypertensive patients with and without TOD.

The most prominent finding of our study was the progressive decline in the AVR across the three groups. Retinal vascular caliber, particularly AVR, is widely recognized as one of the most robust quantitative biomarkers of hypertensive microvascular damage, with AVR values below 0.66–0.75 commonly considered indicative of hypertensive vascular remodeling [14,15,16]. Our findings are consistent with the Beaver Dam and Rotterdam studies, which demonstrated that reduced CRAE and AVR are associated with HT and an increased risk of future cardiovascular events [3,17]. More recently, DL-based studies have confirmed the reproducibility of AVR as a quantitative biomarker, achieving HR classification accuracies exceeding 95% [14,18]. Unlike previous studies primarily focused on HR detection, our cohort was restricted to patients with absent or mild HR and stratified according to extra-retinal TOD. Importantly, the association between TOD and lower AVR persisted in analyses restricted to hypertensive participants after adjustment for age, sex, and HT duration. These findings suggest that AVR is associated with greater systemic vascular burden beyond the severity of clinically visible retinal changes.

Arterial network metrics demonstrated hypertensive microvascular remodeling beyond changes in vascular caliber. Fractal dimension and VAD declined stepwise, whereas reductions in SVD and vessel diameter were mainly observed in patients with HT and TOD; tortuosity increased only modestly. Among hypertensive participants, TOD remained independently associated with lower fractal dimension, VAD, SVD, and vessel diameter after adjustment for age, sex, and HT duration. Previous studies have shown that network-based parameters capture alterations beyond conventional caliber measures, with vascular density, branching geometry, and network complexity emerging as biomarkers of hypertensive vascular injury [16,19]. Consistent with this, recent DL-based retinal vascular analyses have shifted from isolated caliber measurements toward comprehensive vascular phenotyping incorporating VAD, branching geometry, and network characteristics [19,20]. Similarly, in an OCTA study of patients with renal hypertension, the fractal dimension of the superficial and deep macular microvasculature was lower than in healthy controls, although blood pressure was also markedly higher in the patient group [21]. Importantly, these arterial changes were detectable despite absent or mild HR, suggesting potential value beyond conventional qualitative HR grading. In contrast, venous VAD increased overall but showed no consistent stepwise pattern and was not independently associated with TOD, suggesting that arterial network metrics may more closely reflect hypertensive microvascular remodeling.

Venous changes were less consistent. CRVE and venous vessel diameter were higher only in patients with HT without TOD than in healthy controls, whereas venous VAD was higher in the HT with TOD group. Increased retinal venular caliber has been associated with endothelial dysfunction, systemic inflammation, and an increased risk of cerebrovascular disease, all of which are linked to systemic HT [22]. On the other hand, none of the venous parameters remained independently associated with TOD in the adjusted analysis, arguing against progressive venous remodeling with systemic disease burden. This less consistent venous pattern is compatible with findings from Project FOREVER and other population-based studies suggesting that venular caliber shows weaker and less consistent associations with blood pressure than arteriolar caliber [3,17,23]. One possible explanation is that CRVE and network-based venous metrics assess different aspects of the retinal venous circulation [23]. Therefore, the dominant increase in CRVE may indicate that venular caliber is affected earlier or differently than global venous network characteristics in patients with absent or mild HR.

The frequent coexistence of renal, cardiac, and carotid abnormalities highlights the multisystem nature of hypertensive organ damage in this cohort. Recent OCTA studies similarly support a link between ocular and renal microvascular involvement in hypertension; for example, a higher renal resistive index was independently associated with lower deep foveal vascular density [24]. The observed retinal arterial alterations may represent an ocular correlate of systemic vascular injury, supporting further investigation of quantitative retinal assessment as a complement to conventional fundus examination in patients with absent or mild hypertensive retinopathy. In adjusted analyses restricted to hypertensive participants, TOD remained associated with multiple arterial but no venous parameters. This pattern is consistent with the pathophysiology of HT, in which increased vascular resistance, medial hypertrophy, and wall remodeling predominantly affect the arterial circulation. Retinal vascular studies have likewise reported stronger and more consistent associations between arterial biomarkers and hypertensive injury, supporting arterial remodeling as the predominant retinal manifestation of chronic HT [14,16]. The limited venous alterations may reflect the early disease stage of our cohort, as all hypertensive participants had absent or mild HR; this hypothesis requires evaluation across the full spectrum of HR.

Hypertension duration was longer in participants with TOD and was therefore a potential confounder. In analyses restricted to hypertensive participants, however, duration was not independently associated with any retinal parameter, whereas associations between TOD and multiple arterial metrics persisted. These associations persisted after adjustment for reported HT duration, but residual confounding by blood-pressure control and antihypertensive treatment exposure could not be excluded. This is relevant because ocular microvascular measures also reflect systemic hemodynamics: short-term blood-pressure variability on ambulatory monitoring was independently associated with a thinner choroid [25], and the association between the renal resistive index and retinal vascular density was accompanied by higher nighttime pulse pressure rather than by differences in office or 24 h mean blood pressure [24]. Pulsatile hemodynamic load and blood-pressure variability may therefore influence retinal arterial measures independently of established TOD, and neither could be assessed in our cohort. Nevertheless, duration may mediate cumulative vascular injury, and its role cannot be disentangled in this analysis.

From a methodological perspective, our study differs from most previous AI-based studies on HR. Recent DL models have predominantly focused on automated detection or severity grading of HR, often reporting excellent classification performance but providing limited insight into the underlying retinal microvascular alterations [9,26]. Similarly, automated retinal vascular analysis systems have primarily emphasized accurate artery–vein classification and reliable measurement of conventional vascular biomarkers such as CRAE, CRVE, and AVR [15,27]. In contrast, we combined automated DL-based artery–vein segmentation with manual quality control and comprehensive quantitative vascular phenotyping, enabling the assessment of both conventional caliber measurements and arterial network characteristics. More importantly, instead of classifying retinal disease severity, we investigated the relationship between quantitative retinal vascular biomarkers and extra-retinal TOD in patients with absent or mild HR. These findings suggest that quantitative retinal vascular assessment may complement conventional fundus examination by providing information associated with established extra-retinal TOD. Whether these measurements can identify systemic vascular injury before TOD develops remains a hypothesis requiring longitudinal validation.

Several limitations should be acknowledged. First, this prospectively enrolled, single-center study had a modest sample size, limiting its generalizability and precluding organ-specific analyses of heterogeneous TOD manifestations. Second, although the analyses accounted for age and sex, with additional adjustment for HT duration among hypertensive participants, residual cardiovascular confounding remains possible. Data on current office or ambulatory blood pressure, blood-pressure variability, blood-pressure control, antihypertensive medication classes, smoking, BMI, and dyslipidemia were unavailable. These unmeasured factors may contribute to the observed retinal vascular differences, preventing attribution of these alterations specifically to TOD. Although pairwise comparisons within each parameter were Bonferroni-adjusted, testing multiple correlated vascular endpoints may have increased the risk of type I error. Hypertension duration differed between the hypertensive groups; although it was not independently associated with retinal parameters, its potential mediating role cannot be resolved in a cross-sectional design. Moreover, the TOD group included heterogeneous renal, cardiac, and carotid manifestations with varying degrees of multiorgan involvement. The modest sample size limits reliable organ-specific and burden-based analyses; therefore, the findings should be interpreted as associations with composite TOD status. Third, color fundus photography cannot directly assess capillary-level alterations, and pixel-based vascular measurements remain dependent on image acquisition, quality, axial magnification and segmentation performance despite manual quality control. Moreover, image-related exclusions may have introduced selection bias despite similar exclusion rates across clinical groups. The substantial exclusion rate and need for ophthalmologist quality review also limit the generalizability and clinical applicability of the workflow. Finally, longitudinal multicenter studies are needed to determine whether these biomarkers can predict future TOD or cardiovascular outcomes.

In conclusion, DL-assisted quantitative retinal vascular analysis identified predominantly arterial alterations associated with extra-retinal TOD in patients with absent or mild HR. Among hypertensive participants, these associations persisted for multiple arterial parameters after adjustment for age, sex, and HT duration, whereas venous parameters were not significantly associated with TOD in the adjusted models. However, residual cardiovascular confounding prevents attribution of these alterations specifically to TOD. Further studies incorporating comprehensive cardiovascular risk assessment and longitudinal external validation are needed to establish the clinical value of these measurements.

## Figures and Tables

**Table 1:** Demographic and systemic characteristics of the study groups.

Parameter	Normal (Mean ± SD)	HT w/o TOD (Mean ± SD)	HT with TOD (Mean ± SD)	*p*
Age (years)	56.7 ± 8.9	58.1 ± 9.4	59.8 ± 9.9	0.251
Sex (F/M)	27/27	29/25	28/26	0.929
Duration of hypertension (years)	N/A	8.5 ± 2.3	12.6 ± 4.5	<0.001
Urinary albumin-to-creatinine ratio (UACR), mg/g.	11.5 ± 4.2	17.9 ± 5.8	44.8 ± 28.4	<0.001
eGFR (mL/min/1.73 m^2^)	96.2 ± 14.5	89.1 ± 15.5	54.4 ± 10.7	<0.001
Carotid plaque, *n* (%)	0 (0.0%)	0 (0.0%)	22 (40.7%)	<0.001
Carotid intima-media thickness (mm)	0.58 ± 0.11	0.71 ± 0.11	1.08 ± 0.21	<0.001
Left ventricular hypertrophy, *n* (%)	0 (0.0%)	0 (0.0%)	24 (44.4%)	<0.001

HT, hypertension; TOD, target organ damage; eGFR, estimated glomerular filtration rate; N/A, not available; SD, standard deviation. Continuous variables were compared using one-way ANOVA (two-group comparison for hypertension duration: Welch *t*-test); categorical variables using the chi-square test.

**Table 2:** Comparison of retinal vascular caliber and vascular network parameters among the study groups.

Parameter	Normal (Mean ± SD)	HT w/o TOD (Mean ± SD)	HT with TOD (Mean ± SD)	F	*p*
AVR	0.814 ± 0.038	0.740 ± 0.043	0.655 ± 0.042	203.13	<0.001
CRAE, px	22.64 ± 2.29	22.06 ± 2.53	19.02 ± 2.63	33.04	<0.001
CRVE, px	27.81 ± 2.77	29.82 ± 3.07	28.99 ± 2.99	6.35	0.002
Fractal dimension (Art.)	1.553 ± 0.028	1.503 ± 0.042	1.454 ± 0.049	78.59	<0.001
Vessel tortuosity (Art.)	1.29 ± 0.06	1.31 ± 0.07	1.33 ± 0.08	4.35	0.014
Vessel area density (Art.)	0.065 ± 0.013	0.057 ± 0.014	0.025 ± 0.014	129.37	<0.001
Skeletonized vascular density (Art.)	0.019 ± 0.010	0.015 ± 0.009	0.007 ± 0.002	45.54	<0.001
Vessel diameter, px (Art.)	4.12 ± 0.44	4.04 ± 0.51	3.82 ± 0.50	30.48	<0.001
Vessel area density (Vein)	0.061 ± 0.015	0.064 ± 0.017	0.070 ± 0.019	3.89	0.022
Skeletonized vascular density (Vein)	0.018 ± 0.007	0.019 ± 0.008	0.021 ± 0.009	1.95	0.146
Vessel diameter, px (Vein)	5.07 ± 0.58	5.41 ± 0.56	5.23 ± 0.51	5.25	0.006

AVR, arteriovenous ratio; CRAE, central retinal arteriolar equivalent; CRVE, central retinal venular equivalent; Art., arterial; Vein, venous; px, pixels; SD, standard deviation. F and *p* values are from one-way ANOVA.

**Table 3:** Pairwise comparisons of retinal vascular morphometric parameters among the study groups.

Parameter	Normal vs. HT w/o TOD	Normal vs. HT with TOD	HT w/o TOD vs. HT with TOD
AVR	<0.001	<0.001	<0.001
CRAE, px	0.655	<0.001	<0.001
CRVE, px	0.002	0.107	0.474
Fractal dimension (Art.)	<0.001	<0.001	<0.001
Vessel tortuosity (Art.)	0.342	0.012	0.509
Vessel area density (Art.)	0.008	<0.001	<0.001
Skeletonized vascular density (Art.)	0.093	<0.001	<0.001
Vessel diameter, px (Art.)	1.000	<0.001	<0.001
Vessel area density (Vein)	0.999	0.022	0.260
Skeletonized vascular density (Vein)	1.000	0.168	0.675
Vessel diameter, px (Vein)	0.007	0.345	0.267

Pairwise *p* values were adjusted using the Bonferroni correction. AVR, arteriovenous ratio; CRAE, central retinal arteriolar equivalent; CRVE, central retinal venular equivalent; Art., arterial; Vein, venous; px, pixels.

**Table 4:** Multivariable linear regression of retinal vascular parameters by study group, adjusted for age and sex (*n* = 162).

Parameter	HT w/o TOD vs. Normal: β (95% CI)	*p*	HT with TOD vs. Normal: β (95% CI)	*p*	Overall *p* (Group)
AVR	−0.075 (−0.091 to −0.060)	<0.001	−0.161 (−0.177 to −0.145)	<0.001	<0.001
CRAE, px	−0.62 (−1.56 to 0.32)	0.194	−3.73 (−4.68 to −2.79)	<0.001	<0.001
CRVE, px	1.96 (0.84 to 3.08)	<0.001	1.05 (−0.08 to 2.17)	0.068	0.003
Fractal dimension (Art.)	−0.050 (−0.065 to −0.034)	<0.001	−0.099 (−0.115 to −0.083)	<0.001	<0.001
Vessel tortuosity (Art.)	0.019 (−0.008 to 0.046)	0.160	0.039 (0.011 to 0.066)	0.006	0.022
Vessel area density (Art.)	−0.0083 (−0.0135 to −0.0031)	0.002	−0.0405 (−0.0458 to −0.0353)	<0.001	<0.001
Skeletonized vascular density (Art.)	−0.0041 (−0.0071 to −0.0011)	0.008	−0.0141 (−0.0172 to −0.0111)	<0.001	<0.001
Vessel diameter, px (Art.)	−0.09 (−0.27 to 0.09)	0.330	−0.69 (−0.87 to −0.50)	<0.001	<0.001
Vessel area density (Vein)	0.0032 (−0.0032 to 0.0096)	0.326	0.0091 (0.0026 to 0.0155)	0.006	0.022
Skeletonized vascular density (Vein)	0.0008 (−0.0022 to 0.0039)	0.586	0.0027 (−0.0003 to 0.0058)	0.080	0.198
Vessel diameter, px (Vein)	0.34 (0.13 to 0.54)	0.002	0.14 (−0.07 to 0.35)	0.178	0.007

β, unstandardized regression coefficient; CI, confidence interval. Healthy controls served as the reference category. Each row represents a separate multivariable linear regression model with the retinal parameter as the dependent variable and study group, age, and sex as independent variables. The overall *p* value is derived from a partial F-test for the group term (2 degrees of freedom). AVR, arteriovenous ratio; CRAE, central retinal arteriolar equivalent; CRVE, central retinal venular equivalent; Art., arterial; Vein, venous; px, pixels.

**Table 5:** Multivariable linear regression restricted to hypertensive participants, adjusted for age, sex, and duration of hypertension (*n* = 108).

Parameter	HT with TOD vs. No TOD: β (95% CI)	*p*	Per 1-Year Longer HT Duration: β (95% CI)	*p*
AVR	−0.078 (−0.097 to −0.059)	<0.001	−0.0017 (−0.0040 to 0.0006)	0.153
CRAE, px	−3.08 (−4.22 to −1.94)	<0.001	−0.012 (−0.152 to 0.127)	0.863
CRVE, px	−1.17 (−2.51 to 0.16)	0.084	0.056 (−0.107 to 0.219)	0.495
Fractal dimension (Art.)	−0.046 (−0.066 to −0.025)	<0.001	−0.0009 (−0.0034 to 0.0016)	0.492
Vessel tortuosity (Art.)	0.021 (−0.013 to 0.055)	0.218	−0.0003 (−0.0044 to 0.0039)	0.893
Vessel area density (Art.)	−0.0320 (−0.0381 to −0.0258)	<0.001	−0.00011 (−0.00086 to 0.00065)	0.780
Skeletonized vascular density (Art.)	−0.0107 (−0.0136 to −0.0078)	<0.001	0.00014 (−0.00021 to 0.00050)	0.419
Vessel diameter, px (Art.)	−0.58 (−0.80 to −0.35)	<0.001	−0.006 (−0.033 to 0.022)	0.681
Vessel area density (Vein)	0.0029 (−0.0051 to 0.0108)	0.476	0.00073 (−0.00024 to 0.00170)	0.138
Skeletonized vascular density (Vein)	0.0009 (−0.0028 to 0.0047)	0.622	0.00021 (−0.00025 to 0.00067)	0.375
Vessel diameter, px (Vein)	−0.23 (−0.47 to 0.00)	0.053	0.009 (−0.020 to 0.037)	0.552

β, unstandardized regression coefficient; CI, confidence interval. Hypertensive patients without target organ damage served as the reference category. Each row represents a separate multivariable linear regression model including study group, age, sex, and duration of hypertension as independent variables. AVR, arteriovenous ratio; CRAE, central retinal arteriolar equivalent; CRVE, central retinal venular equivalent; HT, hypertension; TOD, target organ damage; Art., arterial; Vein, venous; px, pixels.

## Data Availability

The data presented in this study are available upon request from the corresponding author (A.G.) due to privacy.
